# pytom-match-pick: A tophat-transform constraint for automated classification in template matching

**DOI:** 10.1016/j.yjsbx.2025.100125

**Published:** 2025-05-02

**Authors:** Marten L. Chaillet, Sander Roet, Remco C. Veltkamp, Friedrich Förster

**Affiliations:** a Structural Biochemistry, Bijvoet Center for Biomolecular Research, Utrecht University, 3584 CG Utrecht, the Netherlands; b Department of Information and Computing Sciences, Utrecht University, 3584 CE Utrecht, the Netherlands

**Keywords:** cryo-ET, Tomograms, Particle localization, Identification, Template matching, Volume registration, GPU-acceleration

## Abstract

Template matching (TM) in cryo-electron tomography (cryo-ET) enables *in situ* detection and localization of known macromolecules. However, TM faces challenges of weak signal of the macromolecules and interfering features with a high signal-to-noise ratio, which are often addressed by time-consuming, subjective manual curation of results. To improve the detection performance we introduce pytom-match-pick, a GPU-accelerated, open-source command line interface for enhanced TM in cryo-ET. Using pytom-match-pick, we first quantify the effects of point spread function (PSF) weighting and show that a tilt-weighted PSF outperforms a binary wedge with a single defocus estimate. We also assess previously introduced background normalization methods for classification performance. This indicates that phase randomization is more effective than spectrum whitening in reducing false positives. Furthermore, a novel application of the tophat transform on score maps, combined with a dual-constraint thresholding strategy, reduces false positives and improves precision. We benchmarked pytom-match-pick on public datasets, demonstrating improved classification and localization of macromolecules like ribosomal subunits and proteasomes that led to fewer artifacts in subtomogram averages. This tool promises to advance visual proteomics by improving the efficiency and accuracy of macromolecule detection in cellular contexts.

## Highlights

• pytom-match-pick is a GPU-accelerated software for template matching in cryo-electron tomograms.
• A novel application of a tophat transform on score maps is tested and proven effective at filtering peaks.
• The tophat transform is integrated into a dual-constraint thresholding strategy which improves classification of macromolecules in a simulated dataset.
• In experimental data, the dual-constraint thresholding strategy leads to improved subtomogram averages.

## Graphical abstract

**Figure:**
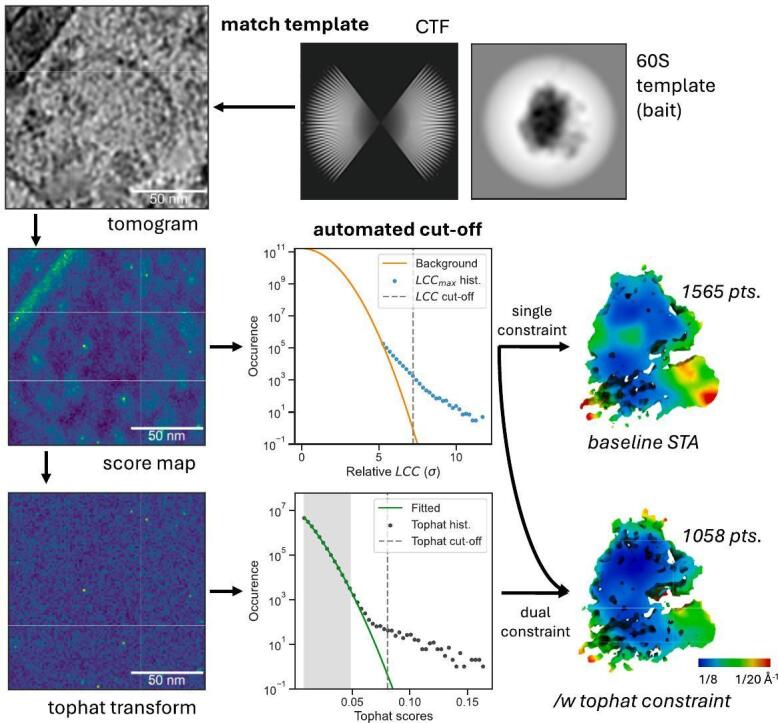

## Introduction

Template matching (TM) enables the *in situ* detection of macromolecules with known structures in three-dimensional (3D) cryo-electron tomography (cryo-ET) data, sometimes also referred to as visual proteomics (Frangakis et al., 2002, Forster et al., 2010). In TM, a template is exhaustively rotated and correlated with the experimental tomograms. Recent advances show that minimally adhering to the angular sampling dictated by the Crowther criterion (Chaillet et al., 2023), coupled with optimizing spatial sampling and band-pass filters enable confident detection of large macromolecules by TM (Cruz-Leon, 2024). These innovations have become available via GPU implementations that allow sufficient translation and rotation sampling at relevant compute time scales (Chaillet et al., 2023, Cruz-Leon, 2024, Maurer et al., 2024). While TM was shown to outperform recent deep-learning (DL) methods for large assemblies such as fatty acid synthase and the nuclear pore complex (Cruz-Leon, 2024), annotations from TM nevertheless typically require further manual curation, region of interest (ROI) selection, and/or threshold selection before being used in subtomogram averaging (STA) procedures.

To improve detection templates need to be weighted with the cryo-ET point spread function (PSF) in the correlation function (Frangakis et al., 2002, Forster et al., 2005). This PSF and its corresponding Fourier transform, the 3D contrast transfer function (CTF), are known to be critical to obtain high resolution in STA (Himes and Zhang, 2018, Bharat et al., 2015), but also improve correlations in TM (Tegunov and Cramer, 2019, Wan et al., 2024). Such 3D-CTF models incorporate dose-weighting, tilt–dependent exposure dampening and CTF parameters, while representing the undersampled Fourier space regions due to the tilting scheme. For TM, it was also reported that the tomogram is ideally reconstructed with phase corrections for the CTF (Tegunov and Cramer, 2019, Wan et al., 2024).

Localization and identification of molecules using TM are often impeded by features in the data with particularly high signal-to-noise ratio (SNR). Such features include strongly scattering contaminations, reconstruction artifacts, or intentionally added gold fiducials for motion registration and targeting. Although the actual shape of these features often varies significantly from the template, their intense scattering results in sharp edges, producing a much higher signal-to-noise ratio (SNR) across all spatial frequencies compared to true positives. True positives display a much lower SNR because biological materials scatter weakly (Reimer and Kohl, 2008). Most TM methods in cryo-EM make use of a locally normalized correlation function, which partly accounts for varying contrast throughout a tomogram but does not fully compensate these SNR differences (Frangakis et al., 2002, Roseman, 2003).

To our knowledge, two background normalization methods have been suggested to reduce false positives in TM score maps, i.e., the map containing the maximum cross-correlation at each position over the rotational search. Firstly, in 2D TM, Rickgauer, Grigorieff and Denk (Rickgauer et al., 2017) suggested a whitening filter which is calculated as the reciprocal square root of the radially averaged power spectrum of the search image (or volume) (Tegunov and Cramer, 2019). This profile is then used as a Fourier filter on both the search image and template, flattening the power spectrum of the image noise and downweighting low-frequency noise that often dominates the signal. Alternatively, Wan, Khavnekar and Wagner (Wan et al., 2024) introduced simultaneous TM of a template and a phase-randomized version of the template in STOPGAP, which essentially correlates the tomogram with a random noise object that afterward is subtracted from the score map of the actual template to reduce background noise. However, both methods reduce correlation with low spatial frequencies and therefore are not suited to fully remove strongly scattering edges that have high SNR over the full range of spatial frequencies. To some extent, the remaining false positives arising from high-contrast artifacts can be filtered out using classification procedures in subsequent STA, but often users are required to manually inspect annotations.

Another subjective issue in TM is that many packages require users to manually specify a correlation threshold to extract candidate particle positions. Only for relatively strongly correlating, abundant macromolecules, such as ribosomes, the true and false positive rate can be approximately derived from the correlation peak histogram and used for threshold estimation (Chaillet et al., 2023). In 2D TM, it has been shown that a threshold can also be estimated by tracking the background variation, with the assumption that the background heavily outnumbers true positives in the position-orientation search space (Rickgauer et al., 2017). The latter approach is also effective for low-abundance macromolecules.

We introduce pytom-match-pick,^i^ an easy-to-integrate open-source command line tool for TM in cryo-ET with GPU acceleration. It supports PSF weighting and different background normalization methods, for which we provide a quantitative comparison of their classification performance. To aid automated extraction, we introduce a morphological operation on score maps, the tophat transform, for removing false positives. We show that the transform can be used to estimate a cut-off useful for constraining annotations and automating picking.

## Implementation

### Calculation of correlation maps

TM in pytom-match-pick bases on a locally normalized cross-correlation of the template with a much larger tomographic volume (Roseman, 2003). The translation search is exhaustive, enabled by Fast Fourier transform, but for orientations dependent on the angular increment between neighboring rotations. As in our previous work (Chaillet et al., 2023), the local correlation coefficient (*LCC)* is defined as a function of the template position, x, (relative to the tomogram) and the template rotation, υ (defined by three Euler angles):

(1) $$\text{LCC}\left(x,\;\upsilon \right)=\frac{1}{P}\sum_{i=\left(1,1,1\right)}^{N_{x}N_{y}N_{z}}\frac{\left(T_{i,\upsilon }*W_{i}-\overset{¯}{T}\right)M_{i,\;\upsilon }\left(V_{i+x}-\overset{¯}{V}\right)}{\sigma_{T}\sigma_{M_{\upsilon }V}\left(x\right)},$$

where *T* and *V* refer to the template and tomogram, respectively, $\overset{¯}{T}$ indicates the mean of the template, $T_{\upsilon }$ and $M_{\upsilon }$ are the template and mask, respectively, rotated to υ, $\sigma_{T}$ is the standard deviation of the template, W is a weighting function and ∗ indicates convolution, $\sigma_{M_{\upsilon }V}$ is the local standard deviation of V under M, and P is the sum of the values in the mask. The summation over i calculates the similarity locally in the tomogram, as indicated by the translational offset, i+x. For efficient implementation, we used the Fourier space definition given in (Roseman, 2003). The maximum value of the *LCC* at each position x in the search volume is given by:

(2) $${\text{LCC}}_{\text{max}}\left(x\right)=max\left\{\text{LCC}\left(x,\upsilon \right):\;\upsilon \in \text{A}\right\}\;,$$

where A is the set of orientations that is searched. For generating angular searches, pytom-match-pick uses the HEALPix library (2024.1) (Górski et al., 2005) to enable on-the-fly generation of angular searches. This method is commonly used in cryo-ET (Zivanov et al., 2022). Additionally, the program automatically calculates the angular sampling based on the Crowther criterion, determined by the particle diameter and the Nyquist or, if specified, low-pass frequency (i.e., target resolution). By default the TM program assumes a spherical mask, this speeds up computation as $M_{\upsilon }$ is identical for each rotation υ, meaning the computationally expensive $\sigma_{M_{\upsilon }V}$ also remains identical. For non-spherical masks a flag can be set to recalculate $\sigma_{M_{\upsilon }V}$ for each rotation of the mask.

The tilt-weighted PSF W is calculated in Fourier space by rotating 2D dose-weighted CTFs to each tilt angle in 3D Fourier space. Tilts are weighted by a dose-dependent B-factor, which changes by −4 Å^2^ per 1 e^-^/ Å^2^, and the cosine of the tilt angle (Bharat et al., 2015, Tegunov and Cramer, 2019). To account for regions in Fourier space where multiple tilts are overlapping, the CTF for each tilt is also weighted by a ramp filter along the y-axis (tilt-axis) that increases linearly from zero to one at the overlap frequency with the closest neighboring tilt.

A simplified binary wedge PSF can also be used for the template weighting, *W*, that creates a binary weighting in the Fourier space region between the minimum and maximum tilt angle (Frangakis, 2002). This binary wedge is multiplied with a single CTF but cannot incorporate tilt-dependent dose or CTF parameters. The defocus value for this single CTF should be set to the first collected tilt-image because this contains the highest spatial resolution as it accumulated the least damage.

A visualization of the two PSFs illustrates that the tilt-weighted PSF models all the undersampled regions in Fourier space and a tilt and dose-dependent drop-off (Fig. S1A). Additionally, the amount of ‘fanning’ of the PSF is dependent on the number of sampling points in Fourier space, which is, in turn, a function of the box size of the template.

### Background normalization of correlation maps

We implemented two normalization methods of the cross-correlation function to reduce false positive detection.

**Whitening filter.** For normalization with a whitening filter, we followed the approach of 2D TM in cisTEM (Rickgauer et al., 2017). First, the radial average of the tomogram’s power spectral density (PSD) is calculated, where the power spectrum is the absolute square of the Fourier transform. The reciprocal square root of this radial profile is calculated, $W=\frac{1}{\sqrt{PSD\left(q\right)}}$, and normalized to a maximum of one to calculate the 1D filter (Rickgauer et al., 2017). The filter is then interpolated to a 3D spatial frequency grid for the template and tomogram to obtain the 3D Fourier space filter. The tomogram is filtered as a preprocessing step, while the template’s whitening filter is multiplied with the 3D-CTF and thus applied during TM.

**Phase randomization.** For normalization in TM with a phase-randomized object, as introduced in STOPGAP (Wan et al., 2024), the random decoy is first calculated by randomly permuting the phases of the Fourier transform of the template up to the Nyquist frequency. During TM the original and randomized templates are both rotated, convolved with the PSF, and cross-correlated with the tomogram (Wan et al., 2024). Their *LCC_max_* volumes and variances are separately computed. Upon completion, the score map of the phase-randomized template is subtracted from the regular score map. The mean value of the phase-randomized *LCC_max_* values is also added back to maintain the background statistics. Unlike in STOPGAP, where phase randomization can be applied mutliple times, pytom-match-pick only provides the option to apply it once.

### Dual-constraint thresholding of particle candidates

Particle candidates are derived from the correlation map based on the peaks of the *LCC_max_*. We propose a dual-constraint strategy to select peaks, firstly based on the value of the peaks and secondly based on the shape.

#### *LCC* background cut-off

Conventionally, the peaks of the *LCC_max_* above a defined cut-off τ assign particle candidates. As a starting point to estimate τ, we adapt the 2D approach from cisTEM (Rickgauer et al., 2017) to 3D:

(3) $$\tau \prime =\sigma \sqrt{2}\;erfc^{-1}\left(2/N_{\mathrm{total}}\right)$$

here, σ is the standard deviation of the background, $N_{\mathrm{total}}$ (${=N}_{\mathrm{voxels}}\times N_{\mathrm{rotations}}$) is the size of the search space, and ${erfc}^{-1}\left(\right)$ is the inverse complementary error function. Thus, τ’ is fully dependent on the search space and the standard deviation. By default, this expression takes the false alarm rate as $1/N_{\mathrm{total}}$, a single false positive over the whole search space. In our approach, we added a false positive ratio (*FP_ratio_)* parameter that allows users to tune the extraction sensitivity,

(4) $$\tau =\sigma \sqrt{2}\;erfc^{-1}\left(2FP_{\mathrm{ratio}}/N_{\mathrm{total}}\right).$$

Increasing the $FP_{\mathrm{ratio}}$ lowers the cut-off and, in turn, increases the number of candidates, on the other hand, decreasing the $FP_{\mathrm{ratio}}$ makes the extraction more restrictive. The effectiveness of the threshold is dependent on the accuracy of the estimated standard deviation of the background.

In pytom-match-pick, we implemented a calculation of the standard deviation of the *LCC* values by tracking it during TM over all rotations and voxels, as suggested in (Rickgauer et al., 2017). For each rotation of the template, after calculating the *LCC* (Equation (1)), the values are squared, summed together, divided by $N_{\mathrm{total}}$, and accumulated into a variable that tracks the variance. After TM finishes, this can be used to calculate σ. Thus, the program precisely tracks the standard deviation of the *LCC* during TM under two assumptions: (1) the background mean is zero and (2) the background heavily outnumbers true positives. During annotation of the *LCC_max_* volume, values are extracted from highest to lowest as long as they fall above the cut-off from Equation (4), while setting values in a radius around each peak to zero to prevent annotating the same particle multiple times. When we only applied this cut-off as a single constraint we refer to it as the ‘baseline’ in the remaining text.

#### Tophat transform constraint

To further constrain annotations and facilitate automation we investigate the use of a tophat transform. The motivation is our observation that high-contrast false positives often produce extended patches of high values in score maps, while true positives tend to show steep local maxima (i.e., sharp peaks) in the correlation map (Cruz-Leon, 2024, Tegunov and Cramer, 2019, Rickgauer et al., 2017). We reasoned that a transformation that can separate extended patches from sharp peaks would be appropriate to improve specificity. Hence, we tested a tophat transform, which can be effective at removing speckles from images in a variety of domains (Dougherty and Lotufo, 2003). While the purpose there is to remove fine features from images, we aimed at the opposite, i.e., filtering out larger features using the tophat transform.

**Fig. 1:**
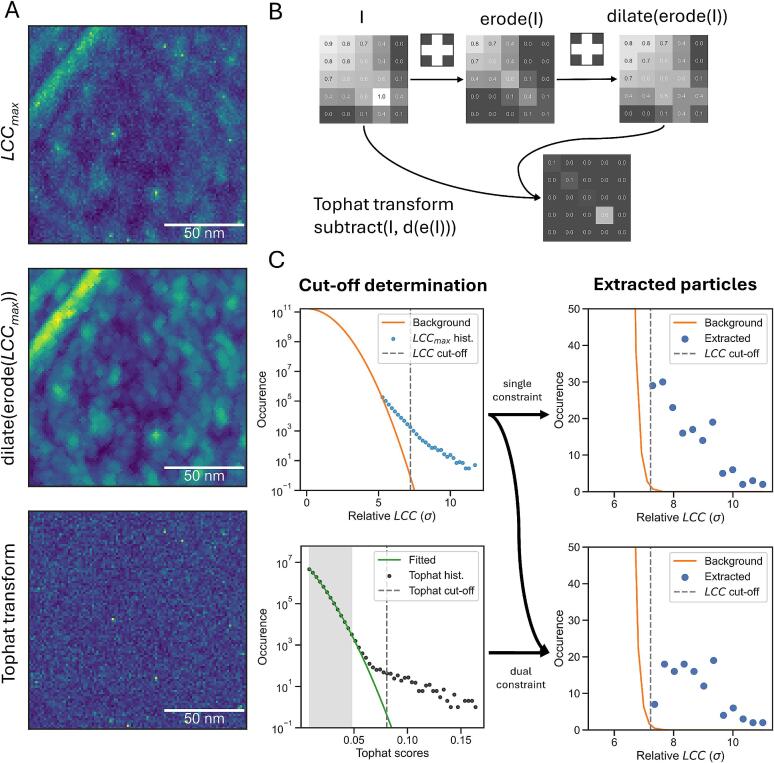
**A tophat transform can reduce background and automate TM annotation.** (**A**) Maximum intensity projections along the z-axis of a score map of 60S LSUs matched with microsome tomograms (top), the morphological opening of the score map (erosion followed by dilation, center), and a tophat transform of the score map (bottom). (**B**) A 2D illustration of a tophat transform on a small image with intensity values annotated in each pixel. The tophat transform involves an erosion followed by a dilation within the specified kernel. The tophat transform is then obtained by subtracting this morphological opening from the original image. (**C**) Automated cut-off estimation in pytom-match-pick. The top left plot shows the default cut-off estimation (see Methods), which determines a threshold (grey dashed line) based on the Gaussian distribution of *LCC* from TM (orange line). The blue dots indicate *LCC_max_* values above the background. The top right shows the distribution of extracted particles after applying the cut-off according to the error function (Equation (3)) and filtering peaks. The bottom left shows the cut-off estimation (dashed line) based on the fitted Gaussian (green) on a histogram of the tophat transform (black dots). Both cut-offs can be simultaneously applied to obtain the final extracted particles that are above both cut-offs (bottom-right). (For interpretation of the references to colour in this figure legend, the reader is referred to the web version of this article.)

A tophat transform is a morphological operation that is applied in real space, which must not be confused with the ‘tophat filter’ which is an unrelated, popular image processing operation in Fourier space. The tophat transform consists of a greyscale erosion around a binary structuring element followed by a dilation around the same structuring element on the eroded volume (this combination is also called ‘opening’). The resulting volume is subtracted from the original volume to obtain the final tophat transform. Fig. 1B illustrates the effectiveness of this transformation in separating a sharp peak from the background based on a defined small kernel. Application of this transform to an *LCC_max_* volume from an experimental tomogram shows that the tophat transform is capable of filtering steep local maxima in TM results (Fig. 1A).

For the implementation, we relied on the SciPy library (1.13) (Virtanen, et al., 2020). The function ‘white_tophat’ from scipy’s ndimage library was used with a footprint created via ‘generate_binary_structure’. The binary structures were 3D and had a connectivity of 1 unless stated otherwise.

Similarly to the *LCC* background cut-off (Section 2.3.1), we applied Equation (4) to find a threshold on the tophat transform of the *LCC_max_* values. Although it is not feasible to track the variance during the rotational search, a histogram of the tophat transform on the *LCC_max_* volume shows a clear Gaussian background after removing zero values (Fig. 1C; bottom left). Based on the log-transform of this histogram, a Gaussian is fitted to the first *N* points where the second derivative is larger than zero.

Both the *LCC_max_* and tophat transform threshold are used as a dual constraint for extracting particles. This dual strategy we further refer to as ‘tophat constraint’. The number of extracted particles — a union of both constraints — is reduced compared to the single constraint baseline as the tophat transform filters annotations. Fig. 1C illustrates the difference in using the baseline cut-off or the dual-constraint strategy.

### Implementation of pytom-match-pick

pytom-match-pick provides a command line interface (CLI) Python package for GPU-accelerated TM (3). The package aims at ease of use while maintaining a simple codebase. Tomograms are provided as MRC files and resulting score maps are written out in the same format (Burnley et al., 2017). Data for the PSF model is extendable based on available metadata: for example, if dose weighting is not to be used, a binary missing wedge in Fourier space can be specified by providing tilt angle limits while providing tilt angles in the IMOD ‘.rawtlt’ or ‘.tlt’ format allows per-tilt weighting (Fig. S1 A; left) (Mastronarde and Held, 2017). Additional defocus files (IMOD’s ‘.defocus’) and accumulated dose (e^-^/A^2^ per line in ‘.txt’) can be provided for CTF and dose-weighting (Fig. S1 A; right). By default, the output is written as a STAR format particle list (Alisterburt et al., et al., 2024), compatible with Warp and WarpTools, and RELION (versions 3 and 4). This enables visualizing the annotations in the napari-based (Sofroniew, 2022) viewer Blik (Gaifas et al., 2024), while results can be used for STA. Additionally, pytom-match-pick integrates with RELION5: it can read tilt-series metadata from a project to generate the PSF and write particle lists with coordinates relative to the tomograms center (Burt et al., 2024).

pytom-match-pick runs on GPU’s using the CuPy library for acceleration (Okuta et al., 2017). Real-spaced input FFT-routines that make use of built-in caching for recurrent calls provide performance, together with the voltools^ii^ library for affine transforms. Supporting functions for calculating filters and particle annotation also make use of the Numpy and SciPy libraries (Virtanen et al., 2020, Harris et al., 2020). The runtime on a tomogram of dimensions 512x512x180 with 50,544 rotations (∼ 7° angular increment) is 353 s. on 2 RTX 2080 Ti. The whitening filter has negligible impact on the runtime as it does not alter the calculation that iterates over the rotations, while phase randomization adds additional steps to this calculation leading to a runtime of 638 s. with otherwise identical parameters. The tophat transform is applied on the extraction step and also does not alter the rate-limiting step of the correlation map calculation and thus has a negligible impact on runtime.

## Results

### A tilt-weighted PSF optimizes correlation

We tested the effect of a tilt-weighted PSF and compared its performance to a binary-wedge PSF with a single defocus estimate using the example of 80S ribosomes associated with vesicles derived from the endoplasmic reticulum (26). This is also the pytom-match-pick tutorial dataset, which we here refer to as DataverseNL-https://doi.org/10.34894/OLYEFI.iii For comparison, we reconstructed the volumes from the tilt series at a voxel size of 13.8 Å and 6.9 Å using novaCTF with phase-flip correction which accounts for the defocus gradient within the reconstructed volume (Methods S2.1 provides a detailed workflow). We expected to find an improvement from the tilt-weighted PSF at a smaller pixel size, as it mainly improves the model for high-resolution information. In contrast, the tilt-weighted PSF leads to a modest but distinct increase in the *LCC_max_* when normalized against the background standard deviation for both reconstructions (σ) (Fig. S1 B and C). Notably, the effect of the pixel size of the reconstruction has a more significant impact on the correlation coefficients of particles, making them easier to distinguish from the background, as also indicated by the increased *LCC_max_* median of candidates. This indicates that TM is still able to exploit resolution in the range of (28 Å)^-1^ to (14 Å)^-1^ in tomograms with either the binary-wedge and tilt-weighted PSF models, and potentially further (Chaillet et al., 2023, Cruz-Leon, 2024). Nevertheless, the 2x upsampling between these reconstructions increases the runtime by a factor of 8. Meanwhile, the smaller pixel size demands increased angular sampling, leading to a further increase in runtime. Overall, we validated our implementation on reconstructions with a 2x magnification difference and showed that the tilt-dependent PSF provides an increase in the similarity score in TM.

### Phase randomization improves 60S ribosome classification

**Fig. 2:**
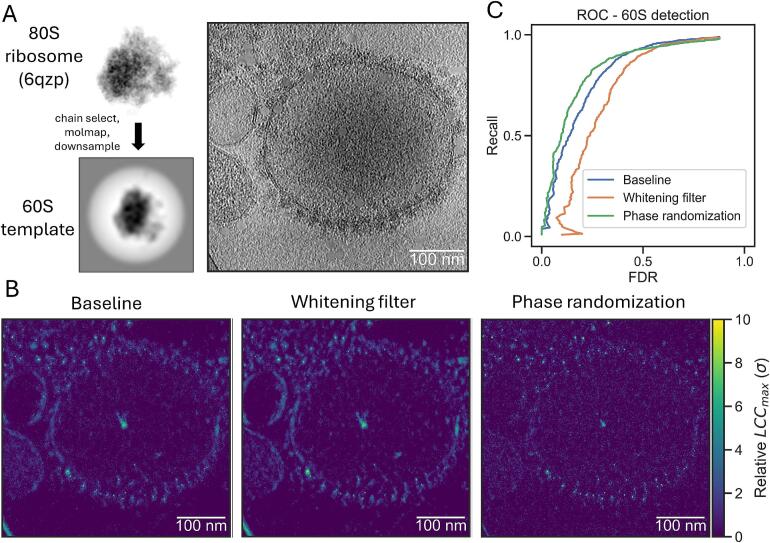
**Background normalization improves TM-based identification for 60S ribosomes.** (**A**) An example of the required input for TM in pytom-match-pick. An atomic model of the 80S ribosome can be used as input from a database. Specific protein/RNA chains can be selected from the model. It needs to be sampled to the spacing of the tomogram and a mask needs to be generated. A tomogram of ER-derived vesicles is used to detect the structure in 3D. (**B**) Illustration of TM score maps (*LCC_max_*) with the 60S large ribosomal subunit for the base method (left), with a whitening filter applied (center) and with flattening via phase randomization (right). The images are maximum projections of the 3D score maps along the z-axis. (**C**) A plot showing the ROC of 60S ribosome detection for the baseline (blue) and two background normalization methods (whitening filter – orange; phase randomization – green). The FDR and recall were calculated over 10 tomograms by comparing them against curated annotations. (For interpretation of the references to colour in this figure legend, the reader is referred to the web version of this article.)

We then compared previously suggested background normalization methods for the classification of the ribosomal large 60S subunit in DataverseNL-https://doi.org/10.34894/OLYEFI (Fig. 2A). Due to its lower molecular weight, the false positive rate is expected to be substantially larger for the 60S subunit compared to the search for the full 80S ribosome. We first ran TM with the 80S ribosome with a 3° angular increment, and manually curated annotations to obtain a ‘ground truth’ list (available in the DataverseNL repository; Methods S2.2). Classification statistics of TM with the 60S ribosome could then be compared with these annotations (Methods S2.3) using a default run (w/o normalization), a whitening filter (Tegunov and Cramer, 2019, Rickgauer et al., 2017), or the phase randomization method (Wan et al., 2024) (Fig. 2).

In the TM score maps the whitening filter enhances sharp edges and membranes, while phase randomization reduces these features compared to the baseline (Fig. 1B). The receiver-operator curves (ROC), which plot the recall against the false discovery rate (FDR), show that phase randomization slightly improves classifier performance, while the whitening filter reduces performance compared to TM without background normalization (Fig. 1C). While all methods level at approximately the same recall, the FDR is increased for the whitening filter leading to worse identification. In contrast, phase randomization reduces the number of false positives.

As the phase randomization method improved TM performance, we additionally tested it on the SHREC’21 benchmark where we calculated per particle ROCs relative to the provided ground truth positions (Fig. S2). In this simulated dataset, it also provided a robust detection improvement compared to TM without background normalization and hence we used it for the remaining sections of this study.

### A spatially restricted kernel for the tophat transform improves classification in SHREC’21

**Fig. 3:**
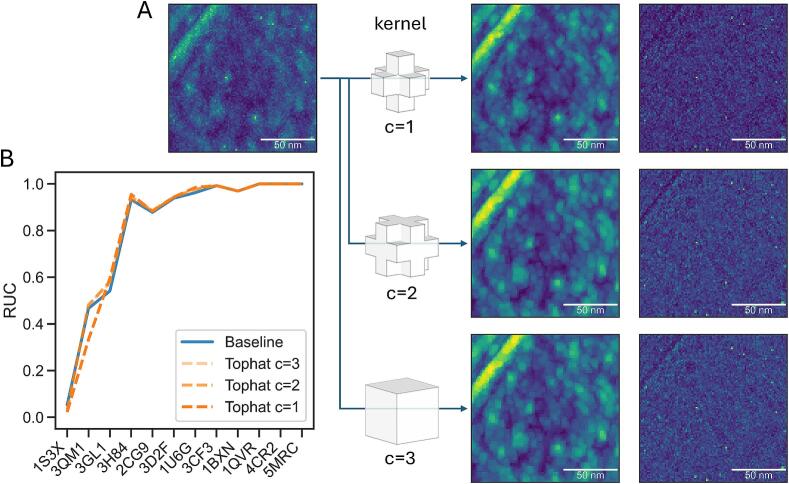
**A tight kernel for the tophat transform provides the best classification in SHREC’21.** (**A**) Visual inspection of the tophat transform using kernels with different spatial extents (or connectivity, denoted by ‘c’ in the figure). The kernels are shown together with their greyscale opening and the final tophat transform. (**B**) Classification results with increasing connectivity (dark to light orange dashed lines) in SHREC’21 compared against the baseline (blue line). The RUC is shown per ground truth particle in the dataset where the particles are ordered by increasing molecular weight from left to right. (For interpretation of the references to colour in this figure legend, the reader is referred to the web version of this article.)

We benchmarked the annotation for the baseline and the tophat transform constraint (dual constraint) on the SHREC’21 dataset. We used the evaluation model (#9) from the competition repository that was reconstructed at a 10 Å voxel size but without any CTF correction. We furthermore evaluated the influence of the spatial extent of the kernel in the tophat transform, testing three kernels with increasing spatial extent (Fig. 3). For the four candidates (baseline and 3x tophat), we calculated ROC curves against the ground truth annotations in the SHREC’21 evaluation tomogram (Fig. S3; Methods S2.3). We note that in some cases the ROC curve has data points with an FDR close one and a low recall, which occurs if the highest-ranking annotation is a false positive such as a gold bead (Fig. S3). Among the different kernels tested the one with the smallest spatial extent (connectivity of 1) has the highest classifier score for the most particles, as estimated by the largest rectangle under the curve (RUC) (Fig. 3B; Methods S2.3). Only for classifying 3QM1 the classifier score is worse, likely due to being too stringent. With increasing spatial extent of the kernel the ROC approaches the baseline performance. Thus, this limited comparison suggests that the tophat transform is most effective with a small spatial extent when it filters for sharp peaks in the score map most aggressively.

### Filtering 60S ribosomal subunits with the tophat transform improves precision

**Fig. 4:**
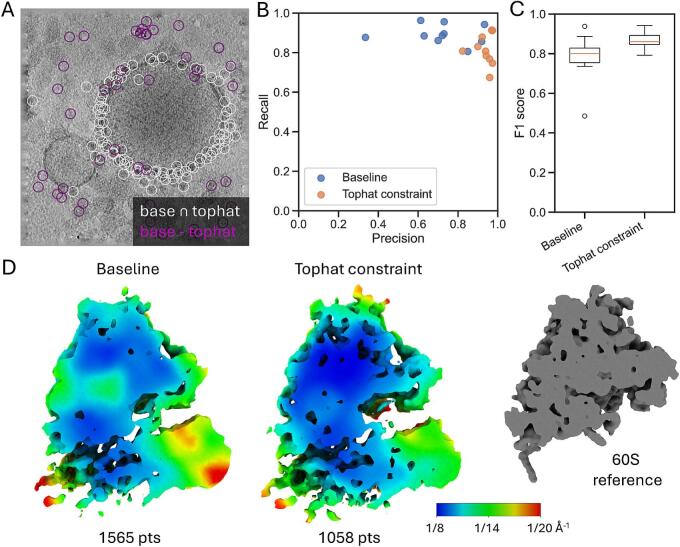
**The tophat constraint improves 60S ribosome annotation in experimental data.** (**A**) Union (white) and exclusion (purple) of the baseline and tophat constraint annotations in a single tomogram from the DataverseNL-https://doi.org/10.34894/OLYEFI. The image is a projection of the tomogram along the z-axis and the circles are also projected from the 3D annotations onto the image. (**B**) Precision and recall of the test dataset for the baseline (blue dots) and tophat constraint (orange dots) annotations, each dot represents the precision/recall on a single tomogram. (**C**) A box plot of the f1-scores per tomogram (N = 10) for the baseline and tophat constraint, where dots indicate points falling outside 1.5 times the interquartile range. (**D**) Local resolution of reconstructions from STA (after alignment). Both methods show structures not present in the original 60S reference (grey structure on the right). The left image shows the local resolution of the baseline set (1565 particles); the right image show the local resolution of the tophat constraint set (1058 particles). The colorbar ranges from (8 Å)^-1^ (blue) to (20 Å)^-1^ (red). The number of particles in each set is noted below the image. (For interpretation of the references to colour in this figure legend, the reader is referred to the web version of this article.)

We further compared the performance of dual-constraint thresholding to the baseline by employing baited reconstruction (Lucas et al., 2023, Hrabe et al., 2012). Here, the search results of the 60S subunit serve as bait for the reconstruction of the complete 80S ribosome from the corresponding subtomograms. Using only this limited bait (Fig. 1A), we can assess possible reference bias based on expected structural features that are absent from the template and should appear upon STA, such as the 40S ribosomal subunit, similar to the originally reported structure (Gemmer et al., 2023). Visual inspection of the annotations in a single tomogram shows that the union of the dual-constraint and baseline annotations is mainly clustered around an ER-derived vesicle, while the unique annotations in the baseline method are also spread around the ice (Fig. 4A). The comparison of the 60S detections with the manually inspected 80S annotations in 10 representative tomograms indicates that the precision improves substantially upon adding the tophat transform constraint at the expense of a small decrease in recall (Fig. 4B). An improvement in the overall performance is reflected in the f1-score, which is the geometric average of recall and precision (Fig. 4C).

To further estimate the quality of both sets, we used STA to resolve the structure and estimated the local resolution. Although both sets show an average containing both the 60S and 40S subunits (confirming our baited reconstruction check), the average derived from the baseline set reaches a maximal local resolution of (8.5 Å)^-1^ (EMD-52598), while the dual-constrained set reaches (7.9 Å)^-1^ with only 2/3 the number of particles (EMD-52599) (Fig. 4D). Additionally, the dual-constrained set reaches higher resolution throughout the stable 60S subunit and part of the 40S subunits. This confirms the reduction of false positives from applying the tophat transform. Overall, dual-constraint thresholding improves the quality of the multi-particle reconstruction.

### Proteasome bait in Chlamydomonas retrieves 26S proteasome with dual-constraint thresholding

**Fig. 5:**
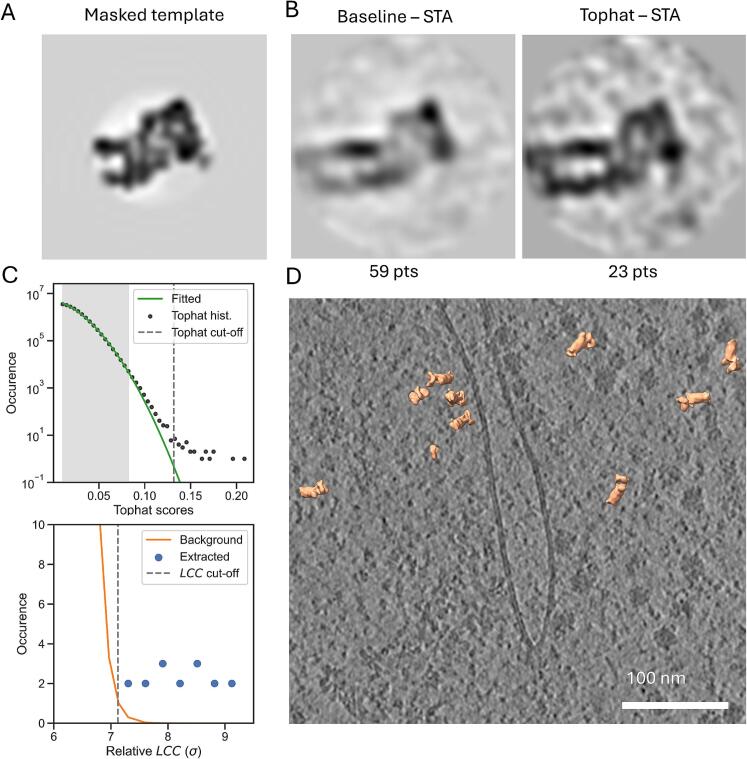
**The tophat constraint improves 26S proteasome classification for *in situ* lamellae.** (**A**) The center slice of the template after multiplication with the generated spherical mask. It illustrates what part of the original structure is removed. (**B**) Center slices of the reconstruction via STA (after alignment) of the baseline set (59 particles) and tophat constraint set (23 particles). Both show recovered structures not present in the initial template. The number of particles in each set is displayed below. (**C**) The top plot shows the Gaussian that was fitted (green line) to the histogram of the tophat transform values (black dots) and the corresponding cut-off (dashed grey line). For fitting the Gaussian only the gray shaded area was used as those points satisfied a positive second derivative. The y-axis shows the log of the occurrence of each bin. The lower plot shows a histogram of the *LCC* (normalized by σ) occurrence of the annotated particles (blue dots) after the tophat constraint. (**D**) A cropped slice of a tomogram focused on a nuclear pore. Particles annotated by the tophat constraint are visualized on top of the slice. (For interpretation of the references to colour in this figure legend, the reader is referred to the web version of this article.)

To further characterize the performance of dual-constraint thresholding we used TM to localize a part of the 26S proteasome in a recently released dataset of *Chlamydomonas rheinhardtii* cells (Khavnekar et al., 2023). Similarly to the ribosome bait, a section of a 26S proteasome, reconstructed with STA, (EMDB-3932) was used as the template by masking (Fig. 5A). The bait contained 50 % of the 20S core particle and one regulatory particle. True positives should all display the full core particle and many also a second regulatory particle associated with it. Three tomograms recorded in the vicinity of the nuclear envelope and ER were used, where proteasomes are likely to localize (Albert, 2017). After TM on tomograms with a voxel size of 15.7 Å, the proteasome annotations of the baseline and dual-constrained set resulted in 59 and 23 particles, respectively. STA of the baseline set gave rise to a structure resembling a 26S proteasome but with clear artifacts, while an STA of the dual-constrained set produced a structure resembling the full 26S proteasome where the 20S core particle and a second regulatory particle is resolved (Fig. 5B).

Based on the STA and the statistics for the cut-off estimation (Fig. 5C), the tophat transform is applicable for a macromolecule that is of markedly lower abundance than the ribosome. Inspecting the proteasome annotations in an abundant section of a tomogram around a nuclear pore, some proteasomes localize close to the pore within the nucleus (Fig. 5D), consistent with previous work (Albert, 2017). Thus, applying a dual-constraint thresholding strategy to TM annotations is applicable for automated localization of a low abundance ∼ 1 MDa complex in full tomograms.

## Discussion

### Closely matching the tomogram and template via PSF modeling

A close match between the target macromolecule in the tomogram and the reference is important for TM. We and others previously showed that angular sampling is a critical parameter determined by the particle diameter and the resolution that is needed for accurate matching (Chaillet et al., 2023, Cruz-Leon, 2024). Furthermore, for a correct match, the CTF needs to be accounted for in the reconstruction of the tomogram and the weighting of the template. Hence, for reconstructions, we here made use of novaCTF, which is able to account for the defocus gradient in tilt-series during tomogram reconstruction. For the template, a tilt-weighted PSF (or 3D-CTF)is more effective than a binary-wedge model with a single defocus value, as also previously demonstrated by others (Tegunov and Cramer, 2019, Wan et al., 2024). We expected a more significant effect of the PSF at smaller pixel sizes (increased spatial sampling) of the reconstruction. However, in the small example shown here, its effect was visible for the low and high-magnification tomograms (with a factor 2 spacing difference). At higher magnification, we did see a clear improvement in the overall similarity score, indicating that the information can be (at least partly) exploited by TM. However, we expect that for higher magnification reconstructions, rather than the CTF model, the quality of tilt-series alignment is a stronger limiting factor for localization. Supporting this, local tilt-series alignment can have a large impact on the *LCC_max_* values in TM, as we previously demonstrated (3), indicating that there might still be a lot to gain in tilt-series alignment. Perhaps, the 3D-CTF might provide more significant improvements at higher magnification in conjunction with local tilt-series alignment.

### Phase randomization improves particle classification

We quantified the effects of a spectrum whitening filter and noise flattening via phase randomization to reduce the response to strongly scattering background objects. In our analysis, phase randomization seems a reliable method for TM that reduced the response to false positives, as indicated by the decreased FDR. On the other hand, spectrum whitening increased the FDR. Earlier results suggested that the whitening filter in tomograms resulted in steeper local maxima for true positives (Tegunov and Cramer, 2019), however, it was not evaluated for classification performance, which we did here. In comparison with 2D TM, where the whitening filtering was reported to improve classification (Rickgauer et al., 2017), the poorer classification performance in tomograms might be due to the reduced SNR in the high-frequency regime of the tomograms compared to 2D data acquired with the entire allowed electron dose. Additionally, in 2D TM, the whitening filter was shown to be effective due to the strong weighting of the high-resolution signal by working with low defocus values. However, data acquisition at low defocus values might not be as beneficial in cryo-ET due to the increased difficulty of aligning the tilt series to a common coordinate system. Our results suggest that spectrum whitening might reduce the classification performance of TM in tomograms, while normalization of the score map via correlation with a phase-randomized template likely improves classification (Wan et al., 2024).

In this work we tested the effect of phase randomization primarily for relatively large targets, the ribosome and the 26S proteasome. For smaller targets with fewer voxels a less variable background correction by averaging multiple noise maps (at the cost of increased computation) might be beneficial.

### Incorporating a tophat transform into an automated thresholding strategy

Annotation via TM in cryo-ET often requires manual inspection, hence we investigated automating the threshold estimation in TM, incorporating a tophat transform to reduce the response to false positives. Morphological operations, such as the tophat transform, can be an efficient tool to post-process TM results. We illustrate that this transform can distinguish sharp peaks in the score map and constrain annotations to steep local maxima. Similarly, Balyschew*, et al.* (Balyschew, 2023) recently proposed post-processing score maps by removing large islands of connected components. However, they did not show to what extent this improves classification by TM. Our results on the SHREC’21 dataset and the ribosomes on ER microsomes show detailed classification statistics that show such methods can be effective.

We incorporated the tophat transform into a dual-constraint thresholding strategy. The first constraint is based on the standard deviation of the background, a method first introduced in cisTEM (Rickgauer et al., 2017), and functions as the baseline extraction threshold. The second is derived from the tophat transform which can be used to find a second cut-off. The combination of these two constraints improves the automated detection of ribosomes and proteasomes, the latter in a cellular environment. The improved precision of the detection is reflected in the sharper subtomogram averages. A downside of these methods is that an additional filtering step by definition reduces particle recall. Secondly, the constraint derived from the tophat transform might put a stronger emphasis on structures in the tomogram that resemble the template as closely as possible while the heterogeneity in the cellular environment can be of interest. While TM is inherently biased to the search structure, the baited reconstruction that we employed, illustrates that the tophat transform does not prevent us from revealing the heterogenous context such as the 40S subunit of the ribosome and the full 20S proteasome barrel. Additionally, by searching for rigid sections of a macromolecule this can be further avoided. Additionally, TM is still often done at relatively low resolution—here we maximally search the structures in resolution up to (30 Å)^-1^—preventing strong bias to conformational heterogeneity. However, it would be good to investigate this further in the future. Overall, although the dual-constraint strategy might remove some recall, it does effectively enable automated annotation for TM in tomograms.

Finally, we did not look into the effect of the width of correlation peaks on the performance of the tophat transform, as might occur for TM on higher magnification tomograms (or misaligned tomograms). However, to indicate the effect, we low-pass filtered a score map with sharp peaks before applying the tophat transform (Fig. S4). The operation seems still capable of filtering the Gaussian smoothed peaks. Thus, we expect that the tophat transform can still be effective for TM at higher magnification.

### Using autocorrelation functions

Although the tophat transform is effective for score map filtering, it only exploits the spatial extent of the *LCC_max_*. A more comprehensive approach would be to exploit both the spatial and rotational aspects of the correlation function. The cross-correlation scores for a given template have a unique spatial and rotational structure defined by its autocorrelation function (Cruz-Leon, 2024). Rather than filtering for steep local maxima, using the autocorrelation function could provide a more precise method for constraining annotations. However, as it is a function of translation and rotation, tracking autocorrelation functions for analysis will require some solutions to work around this multidimensional data.

### Limitations

We used CTF phase-flipping during weighted back projection (WBP) to correct for the defocus gradient in tilt series and improve *LCC_max_* scores in TM. However, other methods often employ CTF multiplication during the reconstruction (Tegunov and Cramer, 2019, Wan et al., 2024, Burt et al., 2024, Turonova et al., 2017). Presumably, in TM, it removes some focus on low spatial frequencies and places stronger weighting on high-frequency regions of the CTF, which might improve precision. Additionally, there are a wide variety of reconstruction methods and filters that could be interesting to compare for TM, for example, Fourier inversion should provide a more faithful reconstruction than WBP. Finally, the effect of denoising algorithms or missing wedge deconvolution on TM is unknown and would be interesting to investigate.

We manually employed baited reconstruction for TM in this paper as pytom-match-pick does not provide a built-in check. It is up to the user to employ this check to test for bias. This can be done by removing certain domains from an atomic model or creating a mask for a density map that focuses on a specific section—the full structure should be retrieved during STA (Yu and Frangakis, 2014).

### Conclusions

Here, we introduced pytom-match-pick, an open-source GPU-accelerated tool for TM in cryo-ET. We demonstrate improvements in TM through tilt-dependent PSF modeling and background normalization techniques. Notably, we introduce a novel approach using a tophat transform to reduce false positives and automate annotation. This method shows particular promise for detecting lower abundance macromolecules like the 26S proteasome in cellular contexts. Overall, pytom-match-pick offers an accessible and effective solution for researchers aiming to locate and identify macromolecules in cryo-ET data, potentially advancing *in situ* structural biology.

## Methods

### Tomographic reconstruction

Raw movies of DataverseNL-https://doi.org/10.34894/OLYEFI were first motion-corrected using MotionCor2 (1.5.0) (Zheng et al., 2017) without assuming any local motion (due to the low SNR of movies in the tilt series). The frames in the repository were already gain-corrected, so this step was skipped. Corrected frames were prepared for AreTomo (1.3.3) (Zheng et al., 2022) by assembling them into a stack (.st), subsequently, a tilt angle file (.rawtlt) was prepared by extracting the angles from the MDOC file. We also created a text file (.txt) with the total dose accumulation per tilt in e^-^/A^2^ using the same ordering as the tilt angle file. AreTomo was then used to create an aligned stack corrected for local motion assuming 5x5 patches. The alignment was optimized for the tilt axis (starting value −88.7°), sample tilt (but not applied to final reconstruction), and an alignment z-height of 1000 voxels (corresponding to ∼170 nm). Dose weighting was also applied in AreTomo by adding the accumulated dose to each row of the tilt angle file, and supplying the software with the pixel size (1.72 Å) and acceleration voltage (200 keV). IMOD (4.10.29) was used to estimate the defocus for each tilt-series with ctfplotter (amplitude contrast 0.08 and spherical aberration 2.7 mm, and 200 keV) (Mastronarde and Held, 2017). It was also used to remove gold beads in the patch-aligned stacks via the programs imodfindbeads and ccderaser. The patch-aligned and gold marker removed tilt-series were then downsampled via Fourier space cropping with IMOD’s newstack by binning 4 times, resulting in a pixel size of 6.9 Å.

The reconstructions were generated using novaCTF^iv^ (Turonova et al., 2017). The stacks were CTF-corrected via phase flipping and 3D reconstructed using weighted back projection, with estimated defocus parameters and a defocus step of 15 nm. The final tomograms had zyx-dimensions of 928x960x500 (xyz-order) at a pixel size of 6.9 Å. Finally, 30 voxels along the x and y edges of the tomogram were tapered using IMOD’s taperoutvol. Tomograms at 8 times binning were also generated from these by Fourier cropping with EMAN2′s e2proc3d.py (2.91) (Chen et al., 2019).

Reconstruction of *in situ* tilt series from *Chlamydomonas rheinhardtii* cells (EMPIAR-11830) used a similar protocol, except motion-corrected stacks were already available in the repository and could be used directly. Alignment of the tilt series was done without local patches and the gold bead removal step was also skipped. The tilt-axis was optimized from an initial value of −270° in AreTomo. These tomograms were exclusively reconstructed with novaCTF to dimensions 1024x1024x300 voxels and a pixel size of 7.8 Å (4 times binning), and EMAN2′s e2proc3d.py was used to Fourier crop them to 512x512x150 voxels and a pixel size of 15.7 Å (8 times binning).

### Manually curated 80S ribosome annotations

To generate ground truth annotations of the 80S ribosome in DataverseNL-https://doi.org/10.34894/OLYEFI, TM was first run with a EM map of the human 80S ribosome (EMDB-2938), downsampled to the tomogram’s pixel size, at a 3° angular increment while providing all the necessary data for a tilt-weighted PSF. Particles were first extracted from the TM results using ‘pytom_extract_candidates.py‘ with an exclusion radius of 8 voxels around each peak. The tomograms and the STAR files with annotations were then opened with Blik to inspect and update annotations (Gaifas et al., 2024). Only minor changes were needed to remove some false annotations and add missed ribosomes, as judged by eye. The manual annotations are also available in DataverseNL-https://doi.org/10.34894/OLYEFI.

### Particle classification

**SHREC’21.** The SHREC’21 dataset was used to assess different spatial extents for the kernel used in the tophat transform (Gubins et al., 2021). Specifically, shrec21_full_dataset_no_mirroring.zip was used from the data repository.^v^ For TM, the available atomic models were downloaded and converted to 3D densities using ChimeraX’s molmap (Meng et al., 2023). Spherical masks were generated for each template that covered the full structure. TM was executed with angular sampling respective to each particle’s diameter. The diameter was chosen along the longest axis for non-globular macromolecules, resulting in the highest possible angle increment for the particle. A binary wedge PSF was used with a defocus estimate of −3.5 μm (300 kV) that was set based on the average defocus range provided in the paper (Gubins et al., 2021). For each particle, we extracted a maximum of 500 candidates with an exclusion radius relative to the particle diameter along its shortest axis, using the default cut-off estimation. For the tophat constraint, the extraction was repeated from the same TM job using different spatial extent for the kernel. All the annotations were then evaluated against the ground truth annotations provided in the SHREC’21 dataset from highest to lowest *LCC_max_* in the list. The FDR, FP/(TP + FP), and recall, TP/(TP + FN), were calculated as a function of *LCC_max_*. The point in the curve where (recall * (1-FDR)) was highest, was taken as the rectangle under the curve (RUC).

**DataverseNL-****https://doi.org/10.34894/OLYEFI**. (This is an excluded set of EMPIAR-11830.) Localization of 60S ribosomes against manually curated 80S ribosome annotations was done to assess multiple TM enhancements. The 60S ribosome template was generated from an atomic structure of the human 80S ribosome (PDB 6qzp) by using large subunit labeled chains. For testing the effects of the binary wedge PSF the tilt angle file was provided with a single defocus estimate (3 μm), while for the tilt-weighted PSF, the respective flag was set together with a text file with tilt-dependent defocus estimates (.defocus) and a text file with the dose accumulation (.txt). The angular sampling increment for TM was set to 3°.

For both background normalization methods, the tilt-weighted PSF was used together with a 3° angular sampling. To compare the effects of background normalization, annotations were extracted from a baseline job and jobs with either of the normalization methods activated. All jobs were forced to extract 500 particles. Similarly to the analysis in the SHREC dataset, the annotations were compared against the curated 80S annotations to analyze the ROC and determine the RUC value.

To compare the effects of the tophat constraint, annotations were extracted from the phase randomization job with either the baseline or dual-constraint threshold while keeping the kernel connectivity at 1, i.e. the kernel with the least spatial extent. For both the baseline and tophat constraint the *FP_ratio_* parameter was kept at its default of 1, and particles were extracted up to the determined thresholds. The recall and precision (TP / (TP + FP)) were calculated only on the full list—not as a function of the *LCC_max_*. The f1-score is calculated as the harmonic mean of precision and recall: 2 * (precision * recall) / (precision + recall).

**EMPIAR-11830.** 26S proteasome localization was performed in 8x binned tomograms reconstructed with novaCTF. For the template, an SPA reconstruction of the yeast proteasome (EMD-6575) was recentered on one of the regulatory caps of the 26S proteasome and then downsampled to 15.7 Å. A mask was created to cover the regulatory particle and 50 % of the 20S core. TM was done with the tilt-weighted PSF and an angular sampling of 7°. Particles were annotated with and without the tophat constraint to compare the effects of the filter.

### STA

Averaging of 60S ribosomes was done as described in Chaillet*, et al.* (Chaillet et al., 2023). Specifically, subtomograms were reconstructed at a voxel size of 3.45 Å and a box size of 120 voxels, wide enough for the reconstruction to cover the small ribosomal subunit and the ER-membrane. Local resolution estimation after refinements was done using RELION’s built-in implementation.

Dynamo was used for averaging 26S proteasomes (1.1.532) (Castano-Diez et al., 2012). Dynamo2m was used to convert STAR files with particle annotations to dynamo tables. The dynamo tables were used to crop subtomogram from the 4x binned reconstruction of novaCTF, using the dynamo software. These were then aligned and averaged for four iterations using the dcp graphical user interface. Aligned particles were displayed on a tomographic slice using ArtiaX (Ermel et al., 2022).

### Visualization and plotting

Local resolution maps of ribosomes were made in ChimeraX (Pettersen, 2021). Other plots and images were made using the matplotlib (Hunter, 2007) and seaborn (Waskom, 2021) libraries in Python.

## Code availability

pytom-match-pick is available on GitHub via: https://github.com/SBC-Utrecht/pytom-match-pick.

## Declaration of generative AI in the writing process

During the preparation of this work the authors used ChatGPT in order to improve paragraph readability. After using this tool/service, the authors reviewed and edited the content as needed and take full responsibility for the content of the published article.

## CRediT authorship contribution statement

**Marten L. Chaillet:** Writing – review & editing, Writing – original draft, Visualization, Validation, Software, Methodology, Investigation, Formal analysis, Data curation, Conceptualization. **Sander Roet:** Writing – review & editing, Software, Methodology. **Remco C. Veltkamp:** Writing – review & editing, Supervision, Methodology, Conceptualization. **Friedrich Förster:** Writing – review & editing, Writing – original draft, Supervision, Resources, Project administration, Methodology, Funding acquisition, Conceptualization.

## Funding

This work was supported by the European Research Council under the Horizon Europe program (ERC Proof of Concept Grant 101113464—CryoET-CryoCloud).

## Declaration of competing interest

The authors declare that they have no known competing financial interests or personal relationships that could have appeared to influence the work reported in this paper.

## Data Availability

All the data and code are publicly available. The manuscript details where it can be found.

## Footnotes

i https://github.com/SBC-Utrecht/pytom-match-pick.

ii https://github.com/the-lay/voltools.

iii https://doi.org/10.34894/OLYEFI, DataverseNL, V1.

iv Commit 4f134c7 on branch ‘master’; https://github.com/turonova/novaCTF.

v https://doi.org/10.34894/XRTJMA.

## References

[b0005] Albert S. (2017). Proteasomes tether to two distinct sites at the nuclear pore complex. *PNAS*.

[b0010] Alisterburt *et al.* (2024) teamtomo/starfile: v0.5.8. (Zenodo).

[b0015] Balyschew N. (2023). Streamlined structure determination by cryo-electron tomography and subtomogram averaging using TomoBEAR. *Nat. Commun.*.

[b0020] Bharat T.A.M., Russo C.J., Löwe J., Passmore L.A., Scheres S.H.W. (2015). Advances in single-particle electron cryomicroscopy structure determination applied to sub-tomogram averaging. *Structure*.

[b0025] Burnley T., Palmer C.M., Winn M. (2017). Recent developments in the CCP-EM software suite. *Acta Crystallogr. D Struct. Biol.*.

[b0030] Burt A. (2024). An image processing pipeline for electron cryo-tomography in RELION-5. *FEBS Open Bio*.

[b0035] Castano-Diez D., Kudryashev M., Arheit M., Stahlberg H. (2012). Dynamo: a flexible, user-friendly development tool for subtomogram averaging of cryo-EM data in high-performance computing environments. *J. Struct. Biol.*.

[b0040] Chaillet M.L. (2023). Extensive angular sampling enables the sensitive localization of macromolecules in electron tomograms. *Int. J. Mol. Sci.*.

[b0045] Chen M. (2019). A complete data processing workflow for cryo-ET and subtomogram averaging. *Nat. Methods*.

[b0050] Cruz-Leon S. (2024). High-confidence 3D template matching for cryo-electron tomography. *Nat. Commun.*.

[b0055] E. R. Dougherty, R. A. Lotufo, *Hands-on morphological image processing* (SPIE press, 2003), vol. 59.

[b0060] Ermel U.H., Arghittu S.M., Frangakis A.S. (2022). ArtiaX: An electron tomography toolbox for the interactive handling of sub-tomograms in UCSF ChimeraX. *Protein Sci.*.

[b0065] Forster F., Medalia O., Zauberman N., Baumeister W., Fass D. (2005). Retrovirus envelope protein complex structure in situ studied by cryo-electron tomography. *PNAS*.

[b0070] Forster F., Han B.G., Beck M. (2010). Visual proteomics. *Methods Enzymol.*.

[b0075] Frangakis A.S. (2002). Identification of macromolecular complexes in cryoelectron tomograms of phantom cells. *PNAS*.

[b0080] Gaifas L., Kirchner M.A., Timmins J., Gutsche I. (2024). Blik is an extensible 3D visualisation tool for the annotation and analysis of cryo-electron tomography data. *PLoS Biol.*.

[b0085] Gemmer M. (2023). Visualization of translation and protein biogenesis at the ER membrane. *Nature*.

[b0090] Górski K.M. (2005). HEALPix:: a framework for high-resolution discretization and fast analysis of data distributed on the sphere. *Astrophys J*.

[b0095] Harris C.R. (2020). Array programming with NumPy. *Nature*.

[b0100] Himes B.A., Zhang P. (2018). emClarity: software for high-resolution cryo-electron tomography and subtomogram averaging. *Nat. Methods*.

[b0105] Hrabe T. (2012). PyTom: a python-based toolbox for localization of macromolecules in cryo-electron tomograms and subtomogram analysis. *J. Struct. Biol.*.

[b0110] Hunter J.D. (2007). Matplotlib: A 2D graphics environment. *Comput. Sci. Eng.*.

[b0115] Gubins, I. *et al.,* 2021. SHREC 2021: Classification in Cryo-electron Tomograms. in *Eurographics Workshop on 3D Object Retrieval*, ed S. a. D. Biasotti, Roberto M. and Lai, Yukun and Rosin, Paul L. and Veltkamp, Remco C. (The Eurographics Association)..

[b0120] Khavnekar S. (2023).

[b0125] Lucas B.A., Himes B.A., Grigorieff N. (2023). Baited reconstruction with 2D template matching for high-resolution structure determination in vitro and in vivo without template bias. *Elife*.

[b0130] Mastronarde D.N., Held S.R. (2017). Automated tilt series alignment and tomographic reconstruction in IMOD. *J. Struct. Biol.*.

[b0135] Maurer V.J., Siggel M., Kosinski J. (2024). PyTME (Python Template Matching Engine): A fast, flexible, and multi-purpose template matching library for cryogenic electron microscopy data. *Softwarex*.

[b0140] Meng E.C. (2023). UCSF ChimeraX: Tools for structure building and analysis. *Protein Sci.*.

[b0145] R. Okuta, Y. Unno, D. Nishino, S. Hido, C. Loomis (2017) CuPy: A NumPy-Compatible Library for NVIDIA GPU Calculations. in *NIPS 2017*.

[b0150] Pettersen E.F. (2021). UCSF ChimeraX: Structure visualization for researchers, educators, and developers. *Protein Sci.*.

[b0155] Reimer L., Kohl H. (2008).

[b0160] Rickgauer J.P., Grigorieff N., Denk W. (2017). Single-protein detection in crowded molecular environments in cryo-EM images. *Elife*.

[b0165] Roseman A.M. (2003). Particle finding in electron micrographs using a fast local correlation algorithm. *Ultramicroscopy*.

[b0170] Sofroniew N. (2022). napari: a multi-dimensional image viewer for Python. *Zenodo*.

[b0175] Tegunov D., Cramer P. (2019). Real-time cryo-electron microscopy data preprocessing with Warp. *Nat. Methods*.

[b0180] Turonova B., Schur F.K.M., Wan W., Briggs J.A.G. (2017). Efficient 3D-CTF correction for cryo-electron tomography using NovaCTF improves subtomogram averaging resolution to 3.4A. *J. Struct. Biol.*.

[b0185] P. Virtanen *et al.*, SciPy 1.0: fundamental algorithms for scientific computing in Python. *Nat Methods* **17**, 261-272 (2020). 10.1038/s41592-019-0686-2 PMC7056644 32015543

[b0190] Wan W., Khavnekar S., Wagner J. (2024). STOPGAP: an open-source package for template matching, subtomogram alignment and classification. *Acta Crystallogr D Struct Biol*.

[b0195] Waskom M.L. (2021). Seaborn: statistical data visualization. *J. Open Source Software*.

[b0200] Yu Z., Frangakis A.S. (2014). M-free: scoring the reference bias in sub-tomogram averaging and template matching. *J. Struct. Biol.*.

[b0205] Zheng S.Q. (2017). MotionCor2: anisotropic correction of beam-induced motion for improved cryo-electron microscopy. *Nat. Methods*.

[b0210] Zheng S.W. (2022). AreTomo: An integrated software package for automated marker-free, motion-corrected cryo-electron tomographic alignment and reconstruction. *J. Struct. Bio.l-X*.

[b0215] Zivanov J. (2022). A Bayesian approach to single-particle electron cryo-tomography in RELION-4.0. *Elife*.

