# Supplementary material for: pytom-match-pick: A tophat-transform constraint for automated classification in template matching
Source: J Struct Biol X. 2025 May 2;11:100125. doi: 10.1016/j.yjsbx.2025.100125 (PMC12139429; doi:10.1016/j.yjsbx.2025.100125)
Supplement: Supplementary Data 1 [file mmc1.docx]

Supplementary Information

pytom-match-pick: a tophat-transform constraint for automated classification in template matching

Marten L. Chaillet^1^, Sander Roet^1^, Remco C. Veltkamp^2^, and Friedrich Förster^1, *^

^1^Structural Biochemistry, Bijvoet Center for Biomolecular Research, Utrecht University, 3584 CG Utrecht, The Netherlands

^2^Department of Information and Computing Science, Utrecht University, 3584 CE Utrecht, The Netherlands

*Correspondence: f.g.forster@uu.nl

#
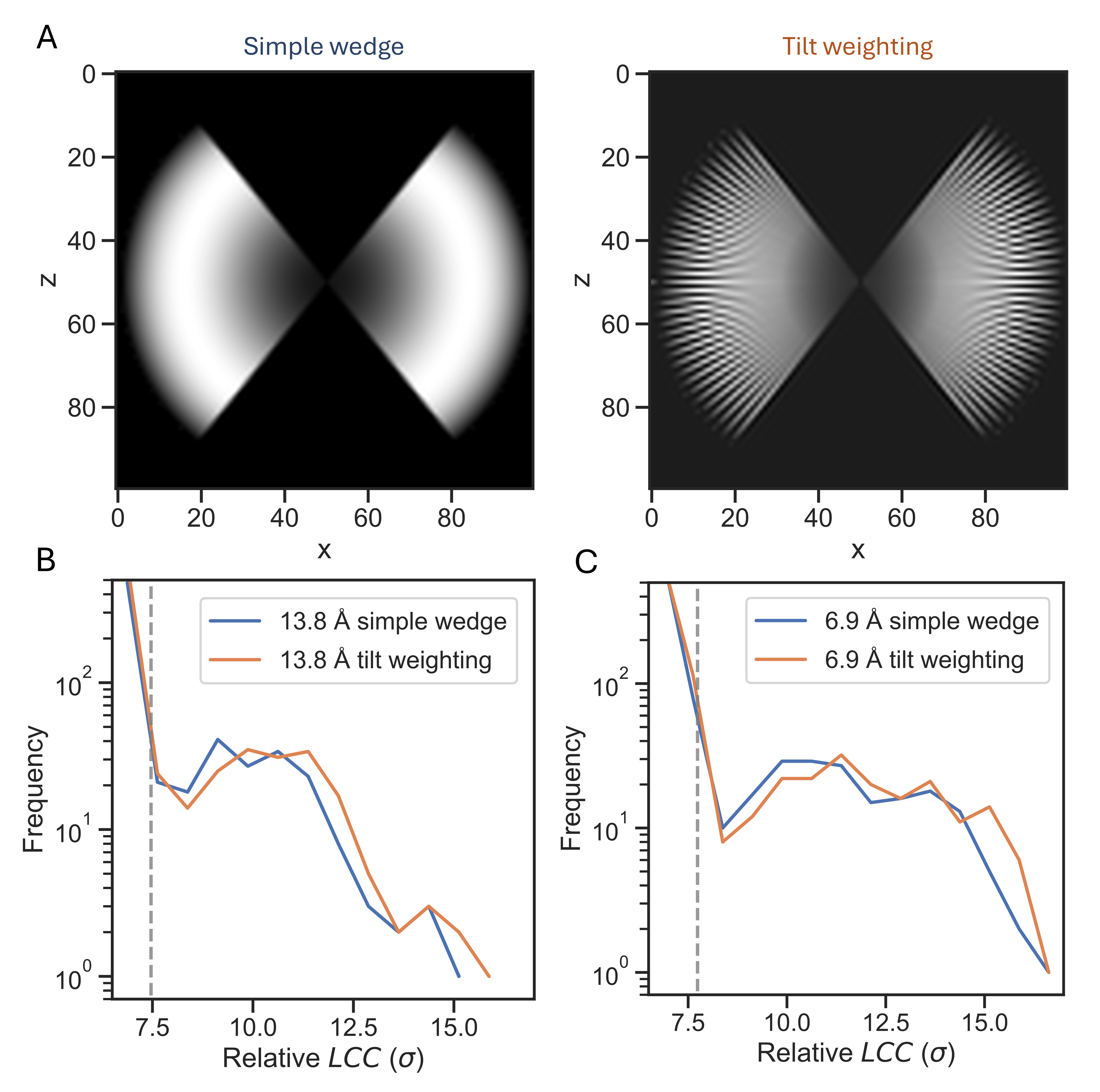
Figures

Figure S1. **3D-CTF models for the tomogram and template improve the cross-correlation.** (**A**) An illustration of two PSF models in Fourier space: the left image shows a binary wedge model with single defocus for the whole tilt-series (simple wedge), and the right shows a weighting scheme that includes tilt-dependent CTFs and dose-dependent B-factor dampening (tilt-weighting). The Nyquist resolution of these models is (26.6 Å)^-1^. Both models are shown as a slice along the y-axis (the tilt-axis). (**B**) Effects of the two PSF models on the correlation in a tomogram downsampled 8 times to a voxel size of 13.8 Å. Each line shows a histogram of the occurrence of the *LCC_max_* of an extracted particle list after division by the standard deviation, σ, as tracked during TM. The blue and orange lines are the simple and tilt-weighting from (A), respectively. The dashed vertical line indicates the cut-off with an expected FAR (= false alarm rate) of 1. This cut-off is identical for each condition due to equal size of the search space and the normalization of the scores with the standard deviation. (**C**) Similar to (**B**) but comparing the *LCC_max_* of extracted particles after TM on a tomogram downsampled to 6.9 Å (orange) voxel spacing with both PSF models.


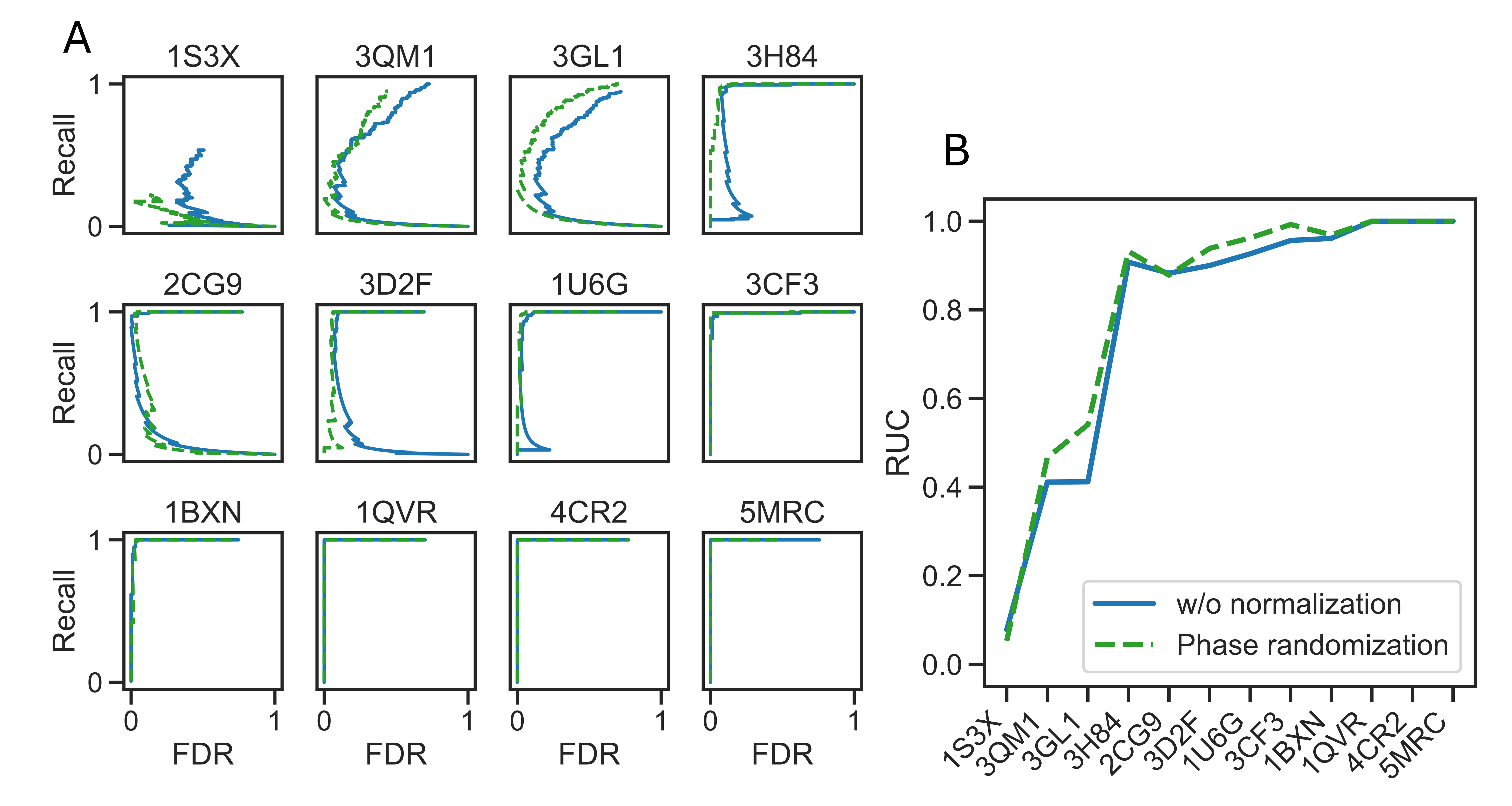
Figure S2. **Per particle ROC for SHREC’21 comparing TM with and without phase randomization.** (**A**) Receiver-operator characteristic (ROC) for each molecule type (identified by protein data bank code) for TM with (green dashed line) and without (blue solid line) the phase randomization method. (**B**) The rectangle under the curve (RUC) for each particle in the dataset which illustrates the optimal classification performance derived from the ROC.


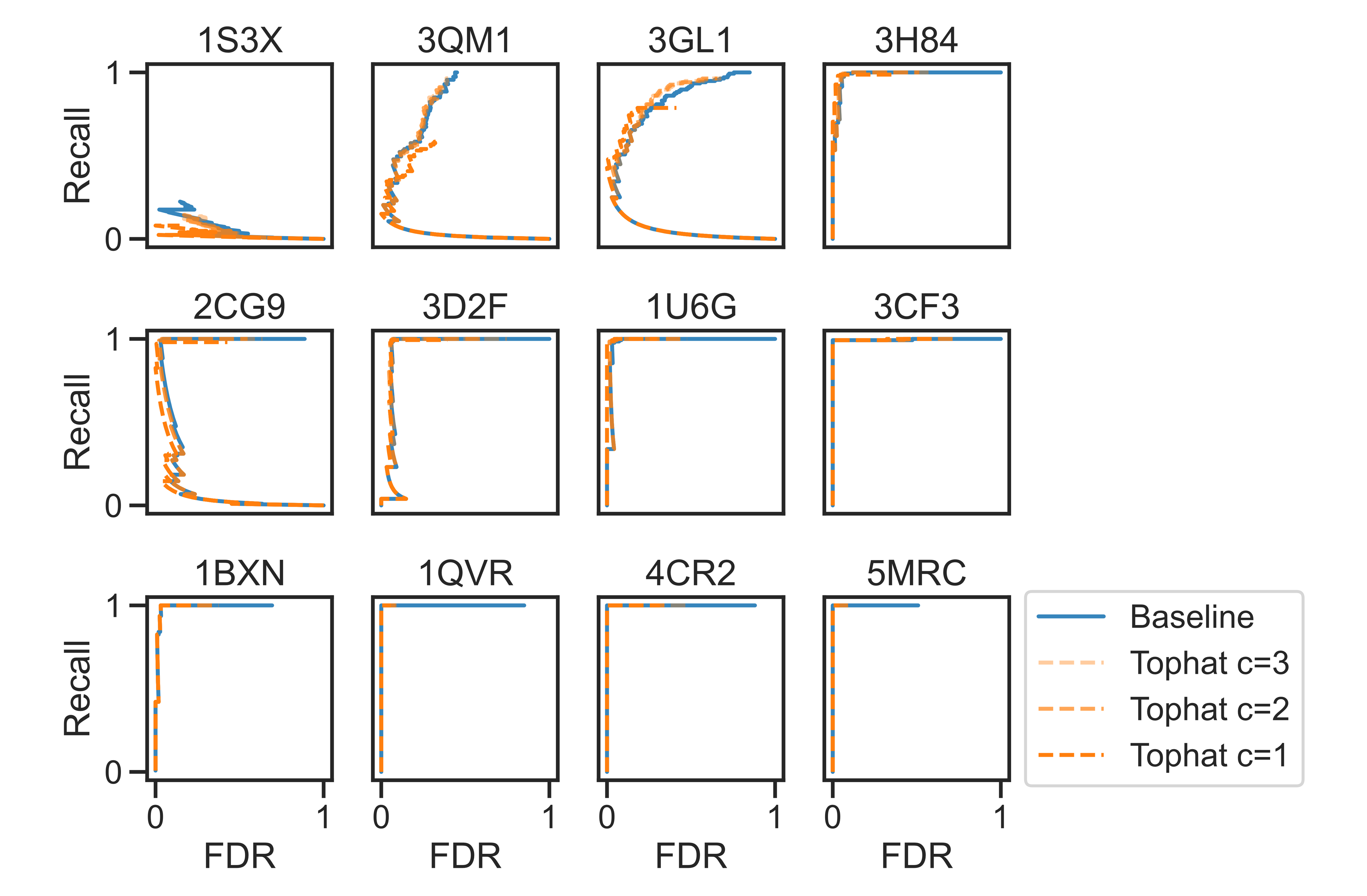
Figure S3. **Per target ROC for SHREC’21 comparing the influence of kernel size on the tophat transform.** For each macromolecule of the dataset, identified by the PDB code, the plot shows four ROCs for the baseline (blue line), and tophat with increasing spatial extents (connectivity) (dark to light orange dashed lines). The x-axis shows the FDR and the y-axis the recall, the panel titles indicate the PDB ID of each particle.


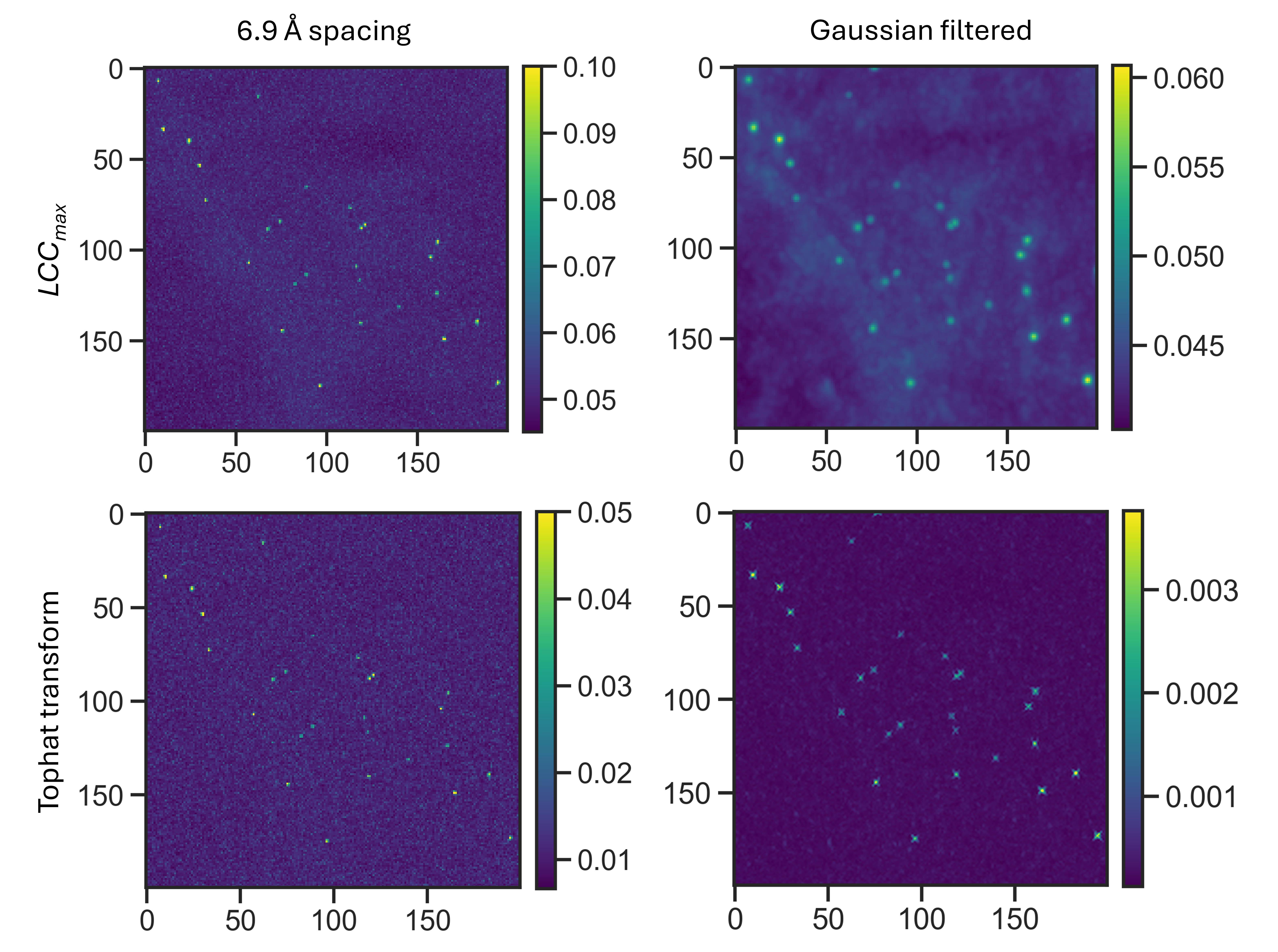
Figure S4. **The tophat transform might also be effective for wider correlation peaks.** The top row shows a maximum intensity projection of a section of an *LCC_max_* volume from 80S ribosome matching on the left (6.9 Å voxel spacing), while the right side shows a similar projection after applying a Gaussian low-pass filter to the volume at (50 Å)^-1^. The bottom row shows the same two volumes after applying the tophat transform with a kernel connectivity of 1.
